# Flow velocity-driven differentiation of human mesenchymal stromal cells in silk fibroin scaffolds: A combined experimental and computational approach

**DOI:** 10.1371/journal.pone.0180781

**Published:** 2017-07-07

**Authors:** Jolanda Rita Vetsch, Duncan Colin Betts, Ralph Müller, Sandra Hofmann

**Affiliations:** 1 Institute for Biomechanics, Swiss Federal Institute of Technology Zürich, Zurich, Switzerland; 2 Department of Biomedical Engineering, Eindhoven University of Technology, Eindhoven, The Netherlands; 3 Institute for Complex Molecular Systems, Eindhoven University of Technology, Eindhoven, The Netherlands; University of Sheffield, UNITED KINGDOM

## Abstract

Mechanical loading plays a major role in bone remodeling and fracture healing. Mimicking the concept of mechanical loading of bone has been widely studied in bone tissue engineering by perfusion cultures. Nevertheless, there is still debate regarding the *in-vitro* mechanical stimulation regime. This study aims at investigating the effect of two different flow rates (v_low_ = 0.001m/s and v_high_ = 0.061m/s) on the growth of mineralized tissue produced by human mesenchymal stromal cells cultured on 3-D silk fibroin scaffolds. The flow rates applied were chosen to mimic the mechanical environment during early fracture healing or during bone remodeling, respectively. Scaffolds cultured under static conditions served as a control. Time-lapsed micro-computed tomography showed that mineralized extracellular matrix formation was completely inhibited at v_low_ compared to v_high_ and the static group. Biochemical assays and histology confirmed these results and showed enhanced osteogenic differentiation at v_high_ whereas the amount of DNA was increased at v_low_. The biological response at v_low_ might correspond to the early stage of fracture healing, where cell proliferation and matrix production is prominent. Visual mapping of shear stresses, simulated by computational fluid dynamics, to 3-D micro-computed tomography data revealed that shear stresses up to 0.39mPa induced a higher DNA amount and shear stresses between 0.55mPa and 24mPa induced osteogenic differentiation. This study demonstrates the feasibility to drive cell behavior of human mesenchymal stromal cells by the flow velocity applied in agreement with mechanical loading mimicking early fracture healing (v_low_) or bone remodeling (v_high_). These results can be used in the future to tightly control the behavior of human mesenchymal stromal cells towards proliferation or differentiation. Additionally, the combination of experiment and simulation presented is a strong tool to link biological responses to mechanical stimulation and can be applied to various *in-vitro* cultures to improve the understanding of the cause-effect relationship of mechanical loading.

## Introduction

Mechanical loading plays an important role in the remodeling of mineralized bone matrix and fracture healing. In healthy bone, the mineralized bone matrix is continuously remodeled, as a function of local mechanical stimuli [1]. In adult bone, osteocytes are considered to be the mechanosensitive cell. They are thought to sense shear stresses (SS) caused by load-induced movement of the interstitial fluid within the lacuno-canalicular system [2]. Stimulation of osteocytes by very high SS leads to the recruitment of osteoblasts to the bone surface and subsequent mineralized bone matrix formation [3]. During fracture healing on the other hand, bone is formed by a cascade of events like gradual stiffening of the forming tissue and tissue deformation. Cells within the repair tissue experience fluid flow as a consequence of loading but the physiological effect of fluid flow is different compared to the effect of fluid flow on osteocytes. Still, the precise role of mechanical stimulation on cells in fracture healing is not clearly understood [4].

In bone tissue engineering (BTE), researchers have been trying to mimic the concept of mechanical loading of bone cells by perfusion flow cultures that are considered to represent the loading concept in bone the closest [5]. Perfusion cultures have been shown to enhance osteogenic differentiation in mouse osteoblast precursor cells and rat bone marrow stromal cells [6–11]. Human derived stem cells cultured under perfusion showed increased levels of osteogenic markers and mineralized matrix deposition [12–16]. Despite these positive effects, it has also been shown that some perfusion culture settings did not support osteogenic differentiation. Very high perfusion velocities led to apoptosis [7, 17], whereas low flow velocities increased cell proliferation instead of osteogenic differentiation [7, 13], and some studies even showed chondrogenic differentiation [18].

At present, a debate exists regarding the role of mechanical stimulation in *in-vitro* BTE cultures. In contrast to mimicking the mechanical loading present in healthy bone, it may be more reasonable to apply a mechanical loading regime that mimics a bone growth and repair environment. Osteocytes were observed starting from day 5 of fracture healing, but with very short and irregularly distributed canaliculi [19]. Based on these observations, it is assumed that precursor cells play a major role in early fracture healing. Preferably, cultures mimicking fracture healing should be performed with precursor cells [4]. Precursor cells, like human mesenchymal stromal cells (hMSCs), have been widely used in the field of BTE [20]. The big advantage of hMSCs is their large proliferation potential, which makes them more suitable for expansion *in-vitro* compared to differentiated cells [4]. Their multipotency, ability to differentiate towards the osteogenic lineage and powerful immunomodulatory effects makes hMSCs also an attractive choice for clinical applications for bone regeneration [21]. Perfusion studies performed with hMSCs showed osteogenic differentiation of hMSCs [13, 16], but very little is known about the loading regime leading to this differentiation.

The mechanical stimulation of cells *in-vitro* depends not only on the mechanical loading regime applied, but also on the scaffold material used. Especially in perfusion cultures, structural parameters of the scaffold like porosity or permeability can have a significant influence on the experimental outcome [22]. Silk fibroin (SF) scaffolds have been widely used for BTE applications [23, 24] due to their excellent biocompatibility [25] and favorable mechanical properties [26]. SF scaffolds have been applied successfully for bone regeneration *in-vivo* [27, 28] and they have been cultured with hMSCs to engineer bone-like tissue *in-vitro* [24].

This study aimed at investigating the effect of two different flow velocities (v_low_ = 0.001m/s and v_high_ = 0.061m/s) on the behavior of hMSCs cultured on SF scaffolds in a perfusion bioreactor. We hypothesized that v_low_ would lead to cell proliferation mimicking the early stage of fracture healing [4], whereas v_high_ would lead to osteogenic differentiation of the hMSCs mimicking bone formation during bone remodeling. Furthermore, we hypothesized that different SS can be defined to lead to different cell behavior for hMSCs. Mineralized extracellular matrix (ECM) formation was assessed by micro-computed tomography (μCT) scans of the *in vitro* cell cultures. SS in SF scaffolds were modeled to estimate the mechanical forces acting on the hMSCs in various locations within the scaffold volume. Subsequently, the modeled SS were mapped to the sites of ECM formation to correlate SS with mineralized ECM formation.

## Materials and methods

### Materials

Dulbecco's Modified Eagle Medium (DMEM), fetal bovine serum (FBS; order number 10270–06), penicillin-streptomycin-fungizone (P/S/F), nonessential amino acids (NEAA), basic fibroblast growth factor (bFGF), β-glycerolphosphate (βGP), ascorbic acid (AA), dexamethasone (Dex), alamarBlue® solution and Quant-iT^TM^ PicoGreen^®^ double stranded DNA (dsDNA) reagent kit were from Gibco (Zug, Switzerland). 1,1,1,3,3,3-hexafluoroisopropanol (HFIP) was from abcr GmbH & Co. (Karlsruhe, Germany). Methanol (MeOH) was from Merck (Zug, Switzerland) and Lithium Bromide (LiBr) from Thermo Fisher Scientific (Reinach, Switzerland). All other substances were of analytical grade and were purchased from Sigma (Buchs, Switzerland). Silkworm cocoons were kindly supplied by Trudel Silk Inc (Zurich, Switzerland).

### Scaffolds

SF scaffolds were prepared as described earlier [23, 29]. Briefly, silk cocoons from *B*. *mori* silkworm were boiled twice for 1h in 0.02M Na_2_CO_3_ and rinsed with ultra pure water (UPW). The silk was dissolved in 9M LiBr and dialyzed against UPW (Slide-A-Lyzer 3.5K MWCO, Thermo Fisher Scientific, Waltham, MA, United States) for 36h, lyophilized for 4 days and dissolved subsequently in HFIP. The resulting 17% (w/v) silk solution was added to 2.5g NaCl of 315–400μm granule size and the HFIP was allowed to evaporate for 3 days. Silk-salt blocks were immersed into 90% MeOH for 30min to induce β-sheet formation [30]. Blocks were air dried over night and NaCl was extracted by immersing in UPW for 2 days. Disc-shaped scaffolds, 8mm in diameter and 3mm in height, were prepared using a razor blade and a biopsy punch. Scaffolds were autoclaved submerged in phosphate buffered saline (PBS) at 121°C for 20min.

### Cell culture and scaffold seeding

hMSCs (Lonza, Walkersville, MD, United States) from human bone marrow aspirate (donor sex: male, donor age: 19, health status: healthy, non-smoker) were isolated and characterized as described before [31]. P3 hMSCs were expanded in expansion medium (DMEM, 10% FBS, 1% P/S/F, 1% NEAA and 1ng/ml bFGF) under standard cell culturing conditions (37°C, 5% CO_2_) for 7 days until about 80% confluence. Cells were resuspended in control medium (DMEM, 10% FBS, 1% P/S/F) at a concentration of 100 million cells per 1ml. 5 million cells were seeded on top of each scaffold by adding 50μl cell suspension by pipetting. Scaffolds were incubated in a 12-well plate for 90min in an incubator. Then, 1ml control medium was added to each well and cells were allowed to attach to the scaffold for 24h. The following day the scaffolds were transferred into the bioreactors.

### Bioreactor culture

**Fig 1:**
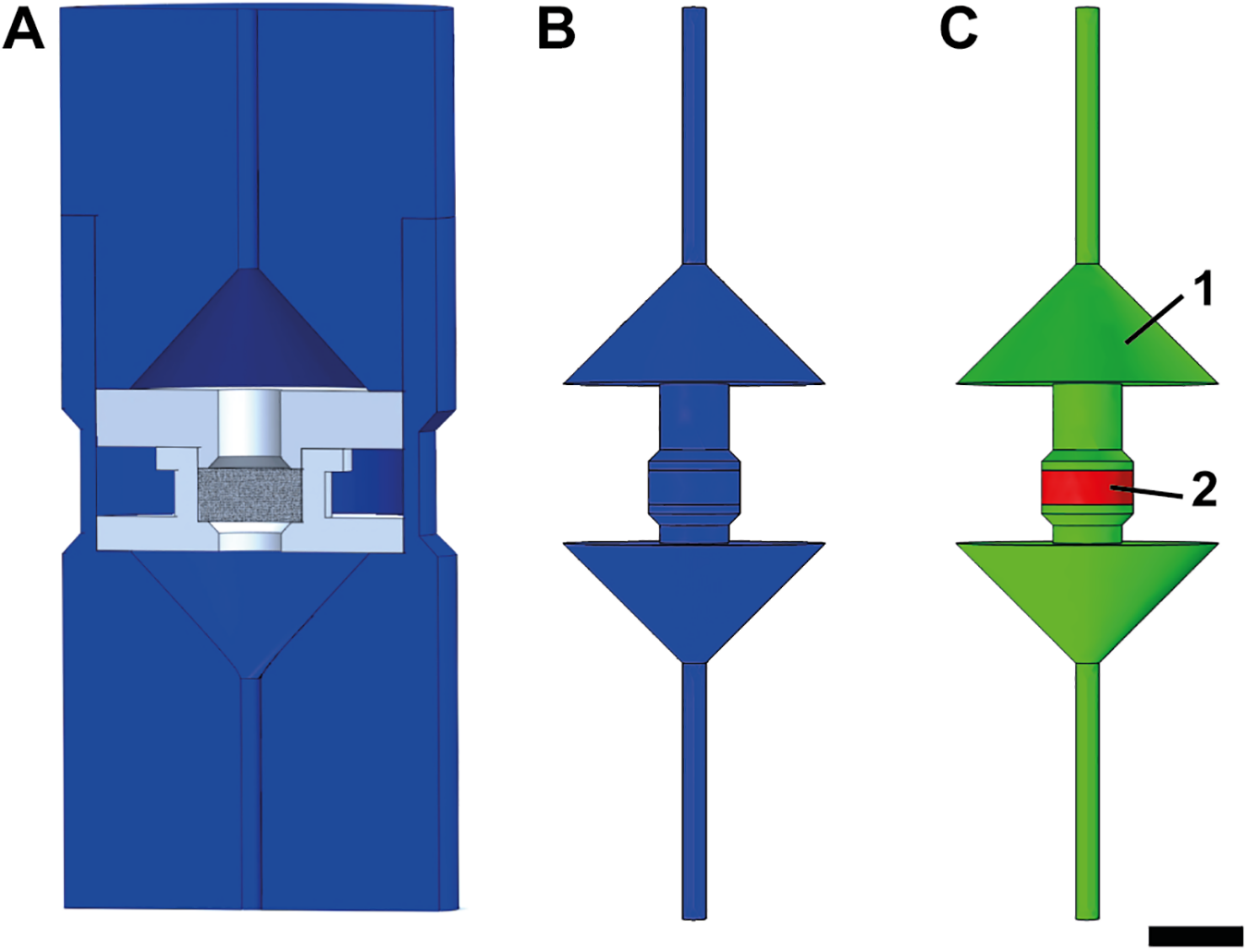
Schematic bioreactor drawings. (A) 3-D computer-aided design model of in-house designed perfusion bioreactor. (B) Inverted volume of perfusion bioreactor. (C) Material definitions for computational fluid dynamics model. (1) Bioreactor: free media flow; (2) Scaffold: porous media flow. Scale bar: 1cm.

Cell seeded scaffolds were cultured in in-house designed perfusion bioreactors (Fig 1A). Bioreactors were provided with 6ml (static group) or 12ml (perfused groups) of osteogenic medium (control medium, 10mM βGP, 50μg/ml AA, 100nM Dex). The medium was replaced 3 times a week by removing 6ml medium and replacing it by either single concentrated osteogenic medium (static group, control medium, 10mM βGP, 50μg/ml AA, 100nM Dex) or double concentrated osteogenic medium (perfused groups, control medium, 20mM βGP, 100μg/ml AA, 200nM Dex) to keep the initial concentration of osteogenic factors constant. The bioreactors were divided into three different groups: (1) low perfusion (v_low_), (2) high perfusion (v_high_), and (3) static (N = 5 per group). The flow rates were set at the pump: Q_low_ = 0.2ml/min for the v_low_ group and Q_high_ = 12ml/min for the v_high_ group, respectively. The bioreactor culture was maintained for 40 days.

### μCT monitoring

Time-lapsed μCT images of all samples (N = 5 per group) were taken once a week over the last 5 weeks of the bioreactor culture (weeks 2 to 6) to monitor 3-D mineralized ECM formation as described before [32]. Samples were scanned in a μCT 80 (SCANCO Medical AG, Brüttisellen, Switzerland) at a voxel resolution of 36μm. Energy level was set to 45kVp and an intensity of 177μA. An integration time of 200ms and 2-fold frame averaging were chosen. Gaussian filtration using a filter width of 1.2 and support of 1 was performed to reduce noise. Mineralized ECM was segmented by thresholding at 97.5mg/cm^3^ hydroxyapatite (corresponding to a grey-scale value of 12.7%). Unconnected particles smaller than 50 voxels were removed from further evaluation using component labeling. The resulting 3-D volume was evaluated morphometrically for mineralized ECM volume (BV) and volume fraction (BV/TV = mineralized ECM volume/total volume), as described previously [33, 34].

### Metabolic activity per cell

Metabolic activity per cell was tested using the alamarBlue® assay at culture week 6. Every scaffold (N = 3 perfusion groups, N = 4 static group) was incubated in 1ml of a 10% (v/v) alamarBlue® solution in control medium for 150min. Fluorescence of supernatant was read in triplicates at an excitation wavelength of 560nm and emission wavelength of 590nm using a plate reader (Tecan Group Ltd., Maennedorf, Switzerland). The fluorescence value of each sample was normalized to the sample’s corresponding DNA amount.

### DNA quantification

After completing the alkaline phosphatase (ALP) assay, scaffolds (N = 3 perfusion groups, N = 4 static group) were left in the 0.2% (v/v) Triton-X-100 solution in aqueous 5mM MgCl_2_ solution and were incubated at room temperature for 48h to make sure the complete DNA content of each scaffold was extracted. The Quant-iT^TM^ PicoGreen^®^ dsDNA reagent kit was used for DNA quantification at culture week 6. After centrifugation, the DNA assay was performed according to the manufacturer’s instructions using the sample supernatant. Fluorescence was read at an excitation wavelength of 480nm and an emission wavelength of 520nm with a plate reader (Tecan Group Ltd., Maennedorf, Switzerland). The amount of DNA per sample was calculated according to the values of a DNA standard curve.

### Osteogenic differentiation

Osteogenic differentiation of the hMSCs was assessed at culture week 6 using a colorimetric ALP assay. Scaffolds (N = 3 perfusion groups, N = 4 static group) were washed with PBS, incubated in 1ml 0.2% (v/v) Triton-X-100 solution in an aqueous 5mM MgCl_2_ solution and disintegrated with a Mini-Beadbeater^TM^ (BioSpec Products Inc., Bartlesville, OK, United States) on ice. After centrifugation at 3000g for 10min the ALP assay was performed with the supernatant. The absorbance was read at 405nm using a plate reader (Tecan Group Ltd., Maennedorf, Switzerland) and the amount of p-nitrophenol per sample was calculated according to the values of a p-nitrophenol standard curve. The ALP value of each sample was normalized to the sample’s corresponding DNA amount.

### Histology

Scaffolds (N = 2 per group) were fixed in 10% (v/v) neutral buffered formalin over night at 4°C and embedded in paraffin. Vertical cross-sections through the middle of the scaffolds were cut to a thickness of 5μm. Hematoxylin & Eosin (H&E) staining was performed to visualize cell nuclei and ECM. Von Kossa (VK) staining was performed to visualize mineralized ECM. Briefly, sections were incubated in a 1% silver nitrate solution (w/w in UPW) and photochemically degraded to silver by exposing to UV light for 45min. The sections were fixed in a 5% silver thiosulfate solution (w/w in UPW) for 2min, dried over night and mounted the following day.

### Computational modeling

Computational fluid dynamics (CFD) analyses were performed using the FPMF module (Free and Porous Media Flow) in COMSOL Multiphysics 4.3b (Comsol Inc., Burlington, MA, United States). The geometry was built from computer-aided drawings of the perfusion bioreactor used for the cell experiments (Fig 1A; SolidWorks 2013, Waltham, MA, United States). The 3-D geometry of the perfusion bioreactor was inverted (Fig 1B) and subsequently imported into COMSOL. The geometry was divided into two different domains: (1) the bioreactor (free media flow) and the (2) scaffold (porous media flow; Fig 1C). The bioreactor was meshed using the built-in mesher optimized for fluid dynamics simulation. A mesh sensitivity study was performed. The convergence criterion applied was defined such that the relative difference of the mean velocity between two consecutive refinement steps was below 2% in the two domains. The scaffold domain was modeled according to Zermatten et al. [35] with a porosity of 55%. The permeability of the scaffold was determined according to the method of Ochoa et al. [36] and was set to 1.76*10^-11^m^2^. No-slip boundary conditions were applied and the fluid was modeled as water at 37°C. Two different inlet velocities were modeled: v_low_ = 0.001m/s and v_high_ = 0.061m/s corresponding to the low and high flow rates set respectively at the pump during the bioreactor culture. Simulation results were correlated with 3-D images from μCT scans of week 6 of the cell culture by performing Receiver Operating Characteristic (ROC). The μCT images were superimposed on the CFD results; SS were identified for every voxel in each scaffold and separated for mineralized and non-mineralized ECM. All scaffolds were processed in a single ROC analysis. By incrementally thresholding SS it was possible to determine the number of correctly classified voxels as mineralized (true positive rate) and the number of falsely classified voxels as mineralized (false positive rate). The ROC curve was calculated for v_high_ only, because no mineralization had formed at v_low_.

### Statistics

**Fig 2:**
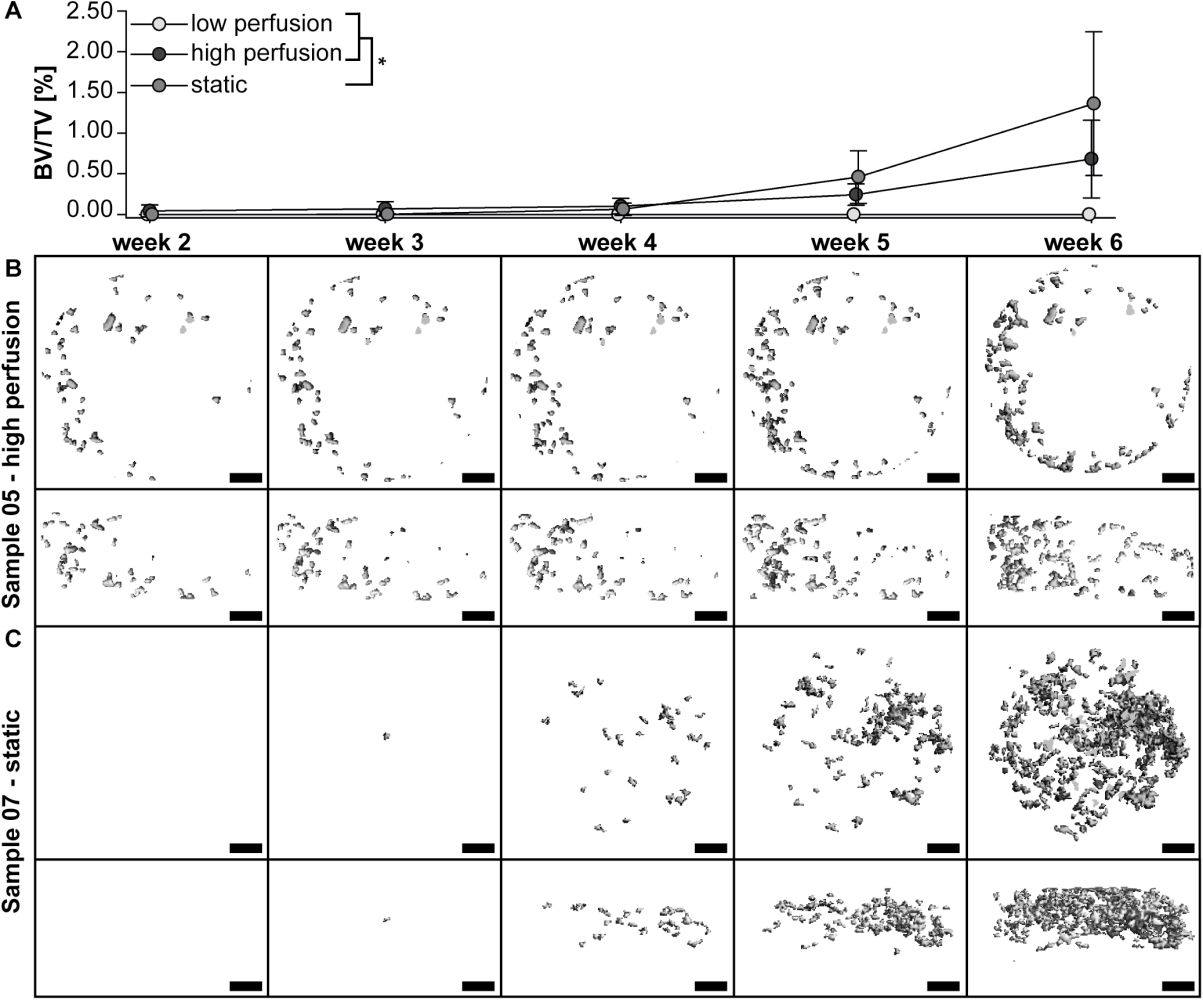
Quantitative data and three-dimensional (3-D) reconstructed images of time-lapsed micro-computed tomography (μCT) scans. (A) Mineralized extracellular matrix (ECM) volume fraction (BV/TV) of one representative sample per group from week 2 to 6. The growth of bone-like tissue was initiated at week 5 of the cell culture in the v_high_ and static group. Images of median sample of (B) v_high_ group (sample 05) and (C) static group (sample 07) shown at each scan time-point. Mineralized ECM grew from the edge towards the middle of the scaffold in the v_high_ group (B), whereas it was formed uniformly throughout the scaffold in the static group (C). Scale bar: 1cm. *p<0.05.

Data was statistically evaluated using PASW Statistics 20.0 (SPSS Inc., Chicago IL, United States). All quantitative data is presented as means ± standard deviation. Biomechanical assay data (metabolic activity per cell, DNA amount, osteogenic differentiation) are represented as fold change of the v_low_ and v_high_ group compared to the static group. Student’s t-test was performed for unpaired and paired data. Comparisons of more than two means were done by an analysis of variance followed by Bonferroni post-hoc corrections. Data was considered statistically significant at p<0.05. ROC curves were calculated using an in-house developed script in Matlab R2014a (MathWorks, Natick, MA, United States). Fig 2 shows upper median samples.

## Results

### μCT monitoring

**Fig 3:**
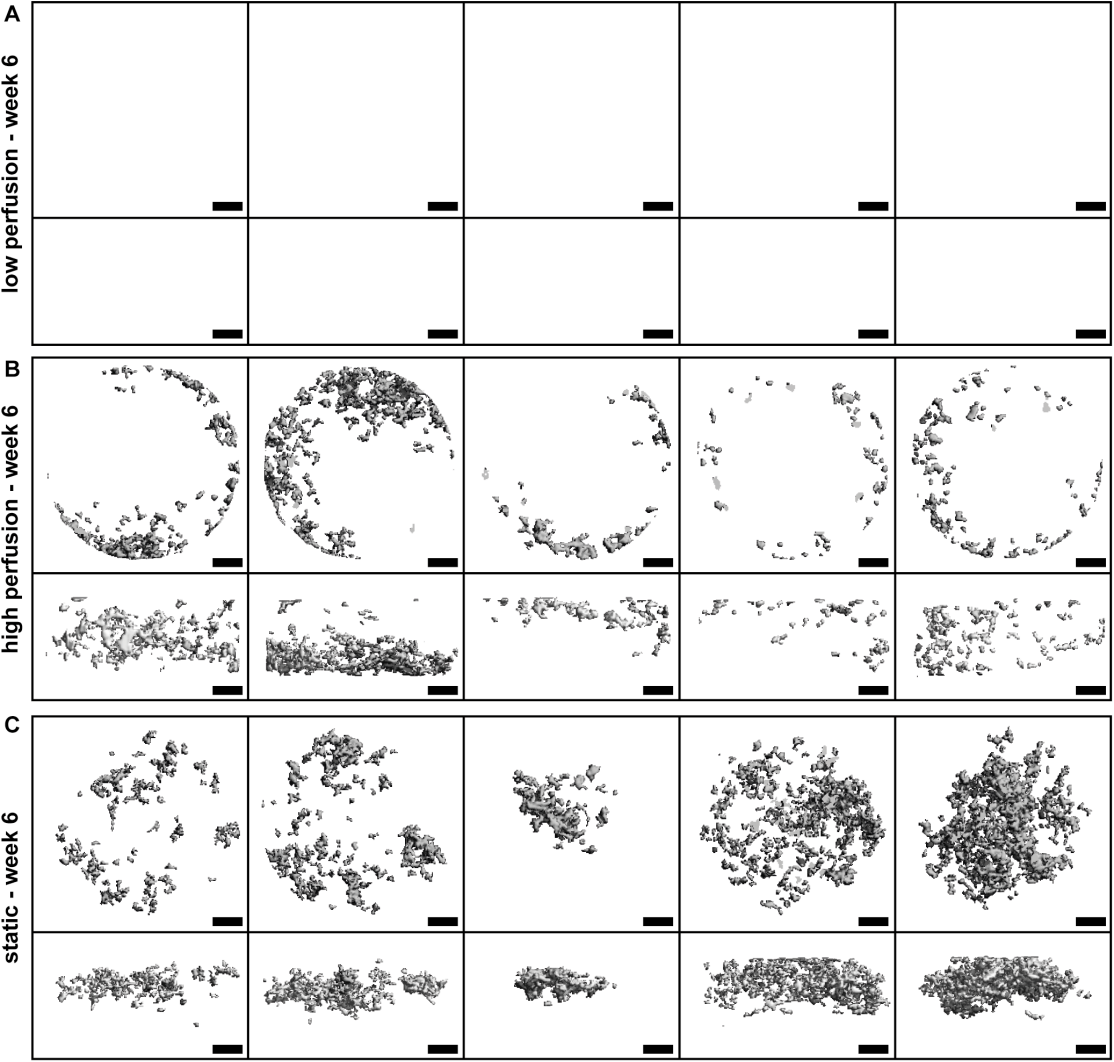
Three-dimensional (3-D) reconstructed images of micro-computed tomography (μCT) scans at week 6 of the culture. Images of all samples of (A) v_low_ group, (B) v_high_ group, and (C) static group. N = 5 per group. Scale bar: 1cm.

None of the samples of the v_low_ group showed any BV formation after 6 weeks of culture (Fig 3A). BV formation was observed for the v_high_ and static group only (Fig 3B and 3C). BV growth was observed starting from week 3 of culture in the v_high_ and static group. Under perfusion, BV was growing from the edges of the scaffold towards the middle of the scaffold (Fig 2B) whereas BV growth was more uniform in the static group (Fig 2C). BV/TV in the v_high_ group increased over time from 0.05% ± 0.07% at week 2 to 0.64% ± 0.45% at week 6 of culture and in the static group from 0% to 1.3% ± 0.88% (Fig 2A). The group was significantly influencing BV/TV, but no differences between all groups could be observed after 6 weeks of culture (p<0.05, Fig 2A).

### Metabolic activity per cell

**Fig 4:**
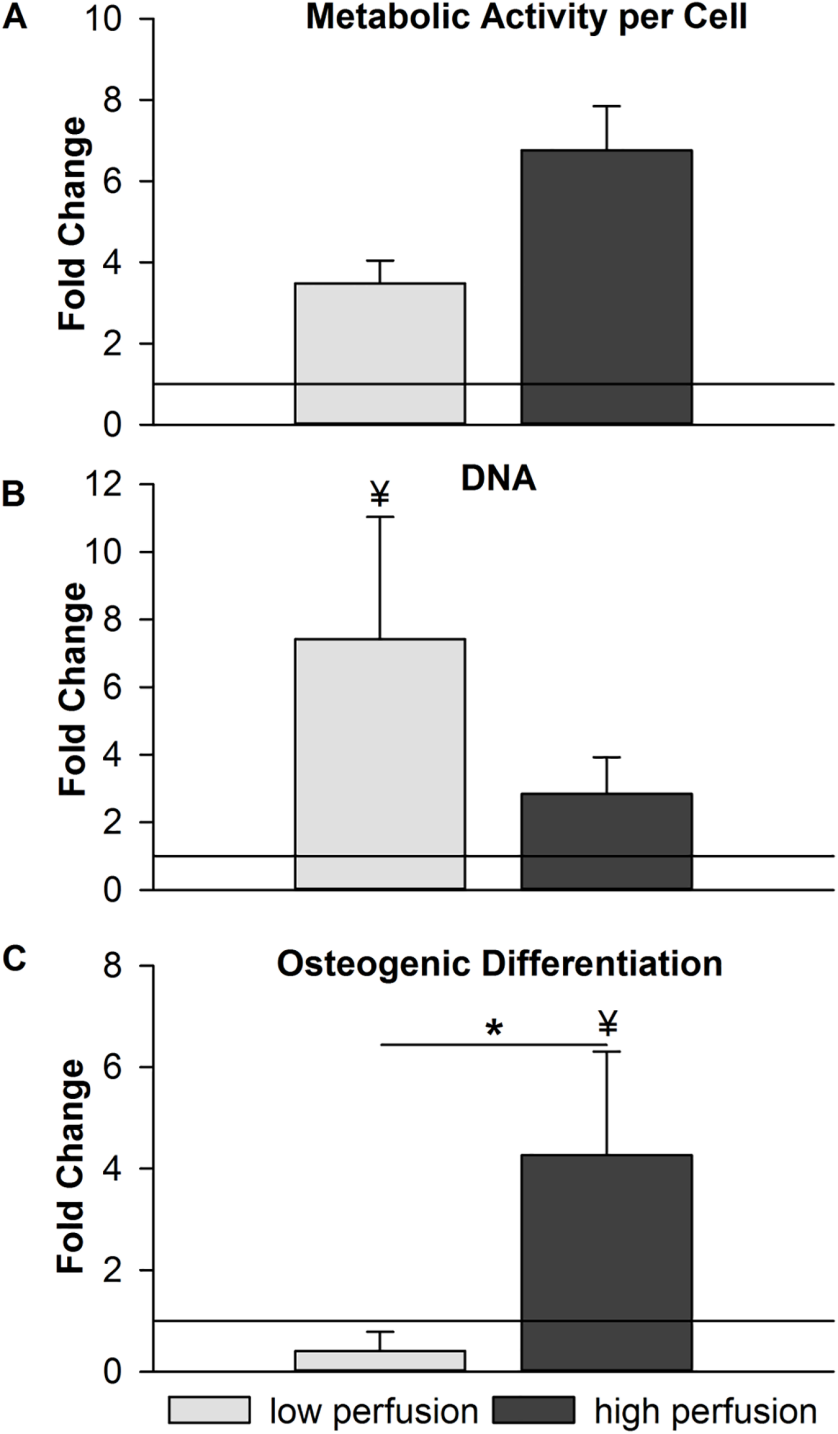
Biochemical assay data at week 6 of culture. Data of the v_low_ and v_high_ group is expressed as fold change compared to the data of the static group. (A) Metabolic activity per cell. (B) DNA amount. (C) Osteogenic differentiation (represented in alkaline phosphatase (ALP) activity). *p<0.05 between v_low_ and v_high_, ¥p<0.05 between v_low_ or v_high_ and the static group. N = 3 perfusion groups, N = 4 static group.

The metabolic activity per cell at week 6 was not significantly different between v_low_ and v_high_ with a trend towards lower activity in the v_low_ group (p = 0.19; Fig 4A). The metabolic activity per cell was increased in both perfusion groups compared to the static group (v_low_: 3.48-fold, v_high_: 6.76-fold), but not significantly.

### DNA quantification

The amount of DNA per sample at week 6 was not statistically different between v_low_ and v_high_ group (p = 0.10) with a trend towards higher cell number in the v_low_ group compared to the v_high_ group (Fig 4B). The DNA amount showed a significant 7.42-fold increase in the v_low_ group and a 2.84-fold increase in the v_high_ group compared to the static group.

### Osteogenic differentiation

ALP activity at week 6 was higher in the v_high_ group compared to ALP activity in the v_low_ group (p<0.05; Fig 4C). Compared to the static group, the ALP activity in the v_low_ group showed a 0.40-fold decrease. In contrast, the v_high_ group showed a significant 4.27-fold increase compared to the static group.

### Histology

**Fig 5:**
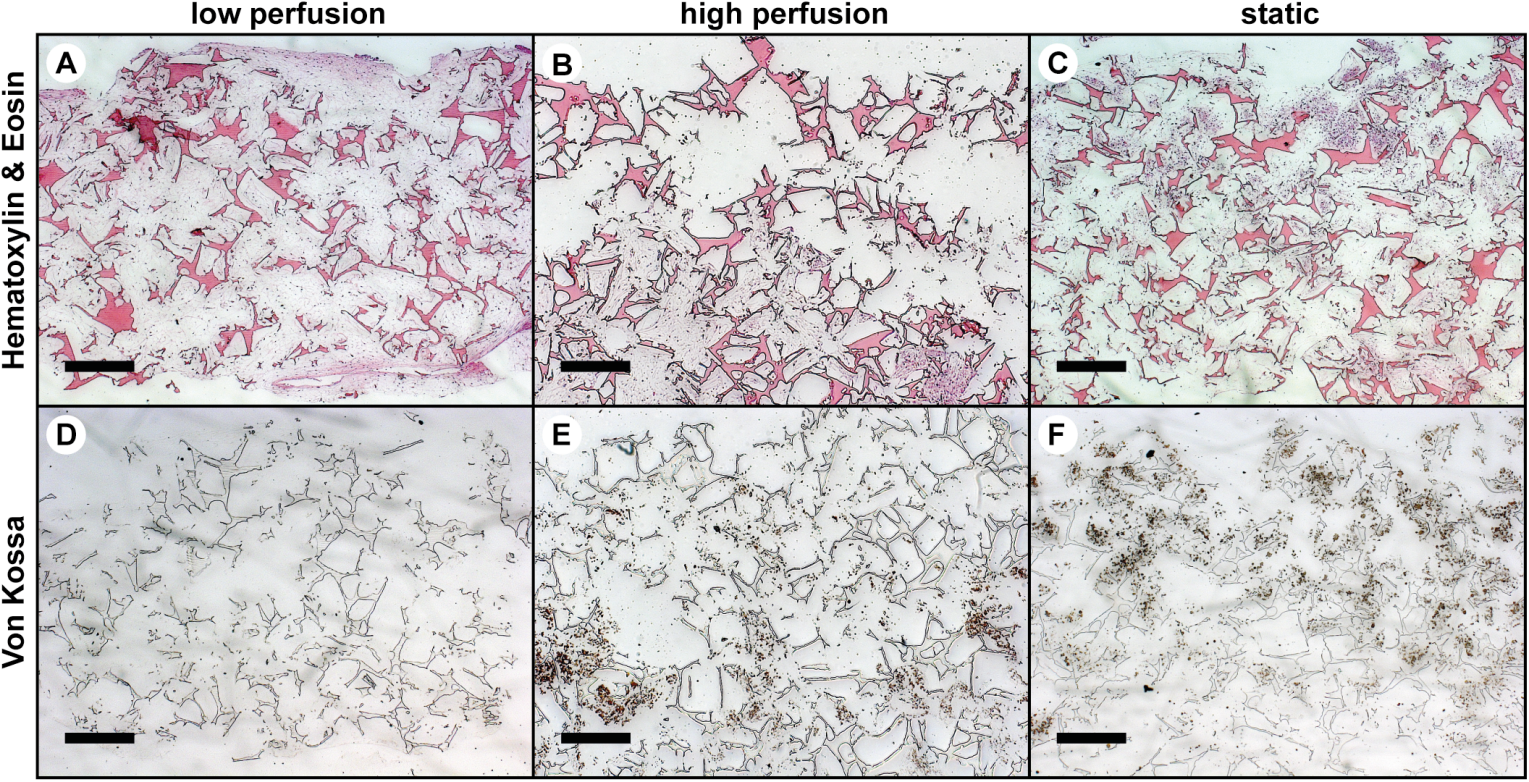
Histological images of vertical cross-sections through the middle of the scaffold at week 6 of culture. (A-C) Hematoxylin & Eosin (H&E) staining. (D-F) Von Kossa (VK) staining. Cells and extracellular matrix (ECM) were uniformly distributed throughout the whole scaffold volume in the v_low_ group (A) compared to the v_high_ group (B) where cells and ECM were more located towards the bottom of the scaffold and static group (C) where cells were more located towards the top of the scaffold. No VK staining was observed in the v_low_ group (D) whereas mineralized nodules were observed in the v_high_ group (E) and in the static group (F) where cells and ECM were present. Scale bar: 500μm.

H&E staining showed a uniform distribution of cells and ECM throughout the whole scaffold thickness for the v_low_ group (Fig 5A), whereas cells and ECM in the v_high_ group were more located towards the bottom of the scaffold volume (Fig 5B) and cells in the static group were more located to the top of the scaffold volume with a less dense ECM (Fig 5C). VK staining revealed no mineralization on scaffolds of the v_low_ group (Fig 5D), but mineralized ECM was observed throughout scaffolds of the v_high_ group (Fig 5E) and in the static group located in the upper scaffold half (Fig 5F).

### Computational modeling

**Fig 6:**
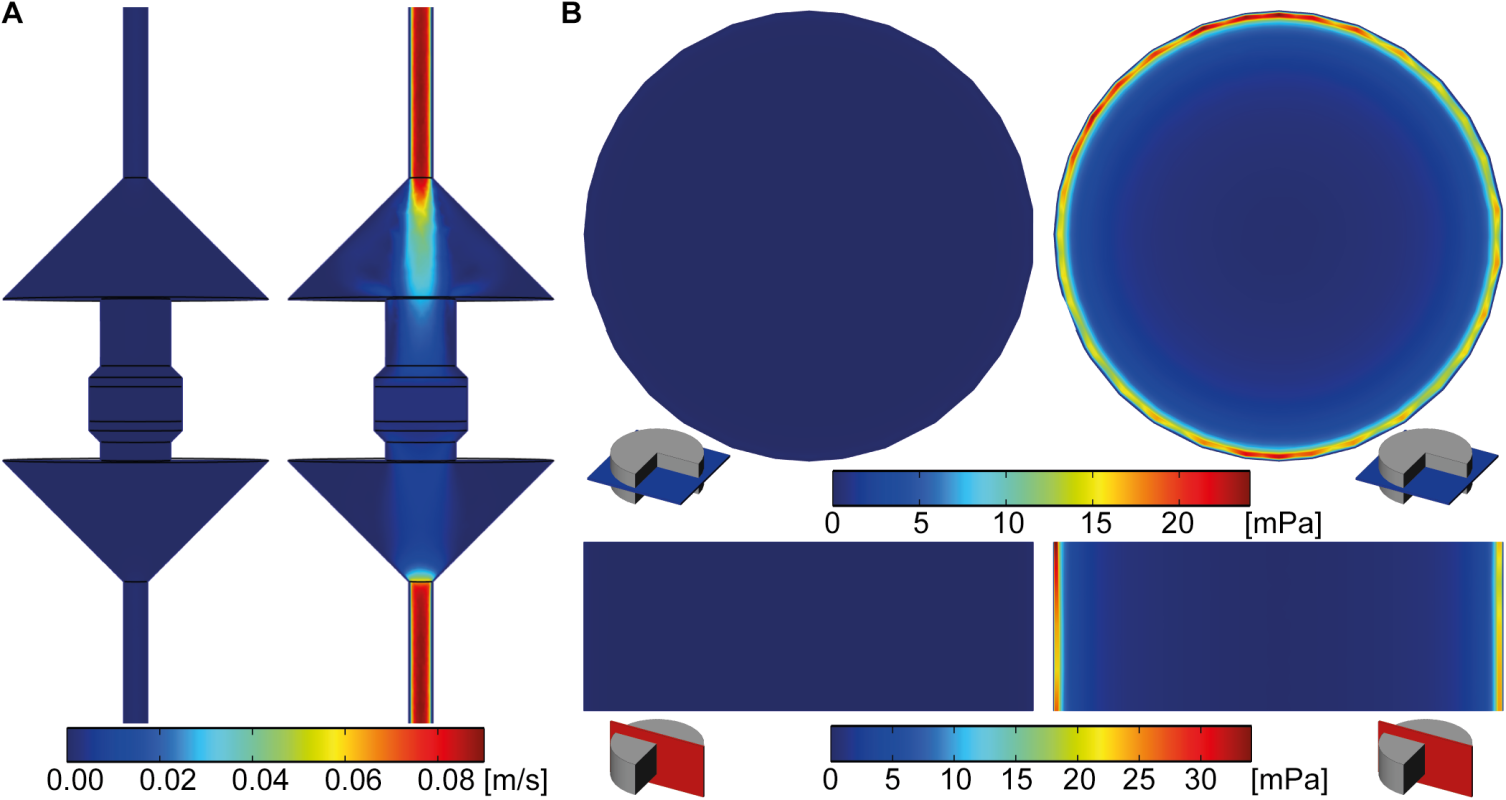
Flow velocity and shear stress (SS) simulation data. (A) Velocity fields of vertical cross-sections at the middle of the bioreactor at v_low_ (left) and v_high_ (right). (B) SS fields of horizontal (blue) and vertical (red) cross-sections through the middle of the scaffold at v_low_ (left) and v_high_ (right). Highest flow velocities were observed at the inlet and the outlet of the bioreactor and highest SS values were observed close to the bioreactor wall in the v_high_ group.

**Fig 7:**
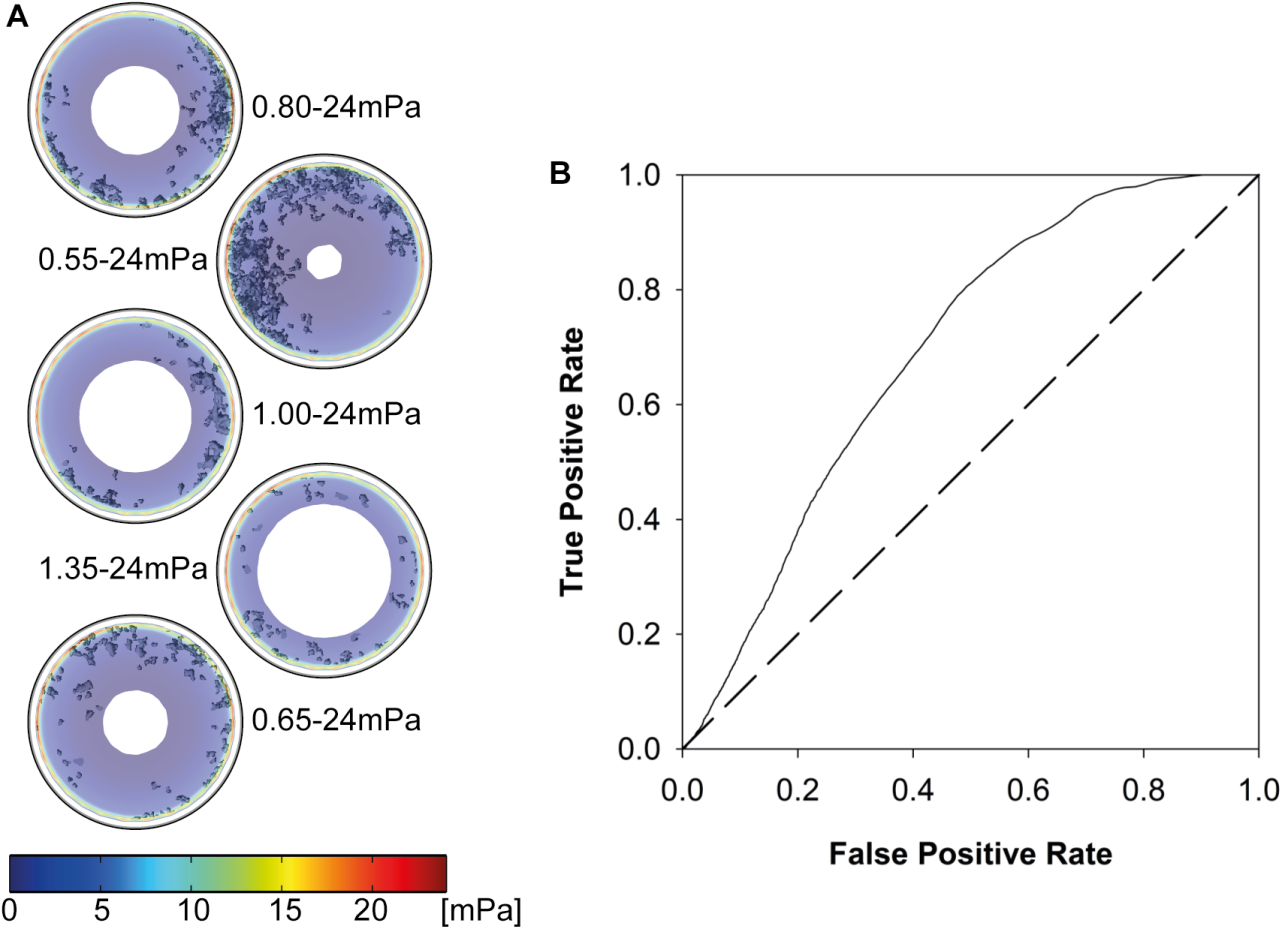
Correlation of simulation results with 3-D images from micro-computed tomography (μCT) scans of week 6. (A) Visual mapping of 3-D μCT data to simulated shear stresses (SS). Mineralized extracellular matrix (ECM) volume was only observed when cells have been subjected to SS of at least 0.55mPa. (B) Receiver Operating Characteristic (ROC) curve for superimposition of 3-D μCT images at week 6 of culture with simulated SS. The curve shows that the SS simulation is distinct from random (random would correspond to 45° line) and the least random SS was observed at 1.47mPa with a true positive rate of 0.81 and a false positive rate of 0.50.

The flow velocity field through the whole bioreactor volume is visualized color-coded for one vertical cross-section through the middle of the bioreactor in Fig 6A. The highest flow velocities were observed at the inlet and outlet of the bioreactor. The maximal flow velocity in the bioreactor at v_low_ was 1.97*10^-3^m/s and 89.23*10^-3^m/s at v_high_. In contrast, the maximal flow velocity within the scaffold was 0.07*10^-3^m/s and 4.46*10^-3^m/s at v_low_ and v_high_, respectively. Maximal SS in the scaffold volume were 0.56mPa at v_low_ and 34.20mPa at v_high_. Horizontal and vertical cross-sections through the middle of the scaffold revealed that the highest SS occurred close to the bioreactor wall (Fig 6B). Maximal SS in these cross-sections were 0.39mPa at v_low_ and 24mPa at v_high_. Visual mapping of SS to 3-D reconstructed images from μCT scans of week 6 showed that mineralized ECM volume was only formed when cells had been subjected to SS in a range of 0.55mPa to 24mPa (Fig 7A). The ROC analysis revealed that the prediction of mineralized ECM is distinct from random with an area under the curve of 0.69. The least random SS (furthest point from the 45° line) for predicting mineralization was 1.47mPa, with a true positive rate of 0.81 and a false positive rate of 0.50 (Fig 7B).

## Discussion

It has been shown that perfusion-induced SS increased osteogenic marker expression and mineralized matrix deposition by human derived stem cells [12–16]. A manifoldness of applied SS can be found in the literature to induce osteogenic differentiation of stem cells, but to date it is still not completely understood how SS affect stem cell behavior exactly. The two different flow velocities applied in the study presented have been chosen to aim at causing biological responses similar to the biological responses occurring during early fracture healing and bone remodeling in healthy bone tissue, respectively. Defining v_high_ and v_low_ based on literature was not possible, due to the wide variety of values reported in literature varying up to a factor higher than 10^3^ [5]. As a consequence, v_high_ was defined based on experiences from previous experiments using our perfusion bioreactor showing mineralized ECM formation at v_high_ (data not shown). V_low_ has been estimated to be 60 times smaller. A dose-dependent differentiation of hMSCs on 3-D matrices in a perfusion setup at flow velocities between 0.1ml/min and 1.5ml/min has been shown before [13]. Nevertheless, the observed results in the current study were surprisingly distinct. μCT monitoring showed no formation of mineralized ECM at v_low_ at all, whereas mineralized ECM formation was observed at v_high_ and in the static group (Fig 3). The evidence of mineralized ECM formation in the static group indicates that the formation of mineralized ECM was suppressed by the application of SS in the v_low_ group. The results observed by μCT monitoring were confirmed by biochemical assays and histology (Figs 4 and 5). These findings reveal a clear dependence of hMSC behavior on the perfusion velocity applied. hMSCs cultured at v_low_ did not differentiate towards the osteogenic lineage although they have been subjected to osteogenic medium. During fracture healing progenitor cells invading the repair tissue are thought to be exposed to mechanical loads stimulating cell proliferation and matrix production [4]. The low flow velocity applied might be able to mimic these mechanical loads of early fracture healing leading to the increased cell number and matrix production observed. From the results displayed, it is not possible to confirm this theory, but it could be tested in future experiments by looking for specific markers of fracture healing.

Cartmell et al. [7] observed similar results. Low flow velocity increased cellular proliferation rate whereas high flow velocity upregulated the osteogenic differentiation potential after 20 days of culture. The comparison to results of other perfusion studies is nevertheless very difficult. Due to different cell types, bioreactor and scaffold geometries used it is difficult to directly compare perfusion velocities and corresponding SS among different studies. The CFD method presented here can serve as a platform to reduce these limitations. The geometry of the bioreactor, porosity and permeability of the scaffold can be included in CFD simulations. SS can then be directly compared between single studies leading to a reduced variation between the different perfusion studies.

In the loaded group, mineralized ECM growth started from the edges and evolved towards the middle of the scaffolds with time. The application of μCT monitoring reveals distinct changes in the very same sample enabling to study the influence of mechanical loading more closely. Based on these results, it can be assumed that the mechanical environment within the scaffold volume changed over time due to ECM deposition and led to this growth pattern. At the onset of the culture, high SS occurred close to the bioreactor wall (Fig 6B). Mineralized ECM started to grow close to the bioreactor wall filling up the pores of the scaffold. Due to the closing of these pores, high SS tend to move towards the middle of the scaffold. Over time, cells cultured closer to the scaffold middle will then be subjected to higher SS leading to subsequent mineralized ECM growth closer to the scaffold middle. Nevertheless–the applied SS of the v_high_ group was not able to increase the amount of mineralized ECM formation over the amount formed in the static group. We assume that there is an optimal perfusion velocity that might maximize the formation of mineralized ECM. While the total amount of mineralized ECM formed is important for bone regeneration, it is of less relevance here, where we were interested in the effects of mechanical stimulation on cellular behavior. Finding an optimal perfusion velocity is challenging due to the irregular pore geometry within the scaffold. Also, the location and spread of each cell influences the load it will perceive. In the model applied here, this effect was averaged by assuming that cells are randomly distributed and regularly spread on the scaffold. In the future, it might be possible to subject the cells to a narrower SS range by applying scaffolds with a smaller pore size distribution and/or more defined pore geometries.

Histology revealed that in scaffolds cultured at v_high_, cells and ECM were preferably located towards the bottom of the scaffold. It is hypothesized that the higher fluid velocity together with gravity forced cells to move towards the bottom of the scaffold. This effect could probably be prevented in future studies by applying oscillatory flow. In the static group, cells were more located towards the top of the scaffold, which is the result of seeding the cells by pipetting on top of the scaffold. This effect might be prevented by dynamic seeding methods that lead to a more even cell distribution. Metabolic activity per cell was not significantly different among the groups and DNA amount was not decreased at v_high_ compared to the static group showing that the high flow velocity did not have a detrimental effect on the hMSCs.

CFD is a useful tool to compute SS in perfused scaffold structures. A variety of simulation techniques have been used from simple analytical to very complex computational models including for example μCT based scaffold structures [37–39]. Several perfusion studies estimated the mechanical loading regime using the cylindrical pore model [7, 8, 37]. However, Jungreuthmayer et al. [38] showed that the model overestimates SS, especially at higher flow velocities. The advantage of the applied simulation model is that it includes the porosity and the permeability of the scaffold used and is still very low in computational costs (less than 1h to solve the model) compared to simulations of real scaffold geometries. Nevertheless, to improve the accuracy of our simulation model the real geometry of the SF scaffolds used should be included. μCT based simulation of SS in scaffolds for tissue engineering has been performed before [38–41]. These studies showed very exact calculations of SS but were mostly limited to a scaffold sub-volume due to limitations in computational costs. Zermatten et al. [41] have been able to simulate SS in a SF scaffold within the same bioreactor geometry as used for this study, but the major drawback of their study was the very high computational cost (several days). Another limitation of μCT-based simulations is that most scaffolds (polymers, gels etc.) are not visible in μCT scans when immersed in culture medium, because they take up the liquid and then their density is not distinguishable from the culture medium. Therefore, the real geometry of the scaffold in culture medium cannot be assessed for simulations and has to be approximated [41, 42]. Also, heterogeneity of the cells needs to be considered. Depending on the differentiation state of the cells, it is possible that some are already differentiated, while other cells are not (yet). Especially cells that are differentiated will be embedded in mineralized ECM over time, resembling osteocytes. These embedded cells will not be able to feel SS to the same extent compared to cells directly on the surface of the tissue or scaffold. Therefore, ECM should additionally be taken into account in simulations, which will increase the complexity of simulations even more.

CFD simulations showed no overlap of SS present at v_low_ and v_high_. Given our data, maximal SS at v_low_ can be considered too small for the induction of osteogenic differentiation of hMCSs. Visual mapping of SS to 3-D μCT images from week 6 of the cell culture points towards optimal SS ranging from 0.55mPa to 24mPa for osteogenic differentiation of hMSCs (Fig 7A). The ROC analysis showed that there is a quantifiable link between areas of high SS and mineralized ECM. The volume of the SF scaffold was not taken into account in the ROC analysis because it could not be distinguished from the culture medium in the μCT scans due to its low density. This then led to an artificially high number of non-mineralized voxels. This effect is small as the scaffolds are highly porous (~90%), but could have increased the number of false positives in the ROC analysis by the volume fraction of the scaffold phase.

SS that induced mineral deposition in this study are similar to values observed in other *in-vitro* cultures [9, 13, 42]. Increased mineral deposition of bone marrow stromal cells was observed for a SS ranging from 10mPa to 30mPa compared to samples cultured at SS smaller than 10mPa or static samples [9]. Similarly, proliferation and metabolic activity per cell of osteoblasts were increased at 0.05mPa compared to samples cultured at higher SS or under static conditions. Subjection of cells to SS higher than 1mPa led to an upregulation of osteogenic differentiation markers [42]. Higher calcium deposition of hMSCs was observed at SS between 0.01mPa and 12mPa compared to samples cultured at SS below 0.01mPa [13].

In the future, ECM growth over time could be included in the simulations as well. It is known, that SS in a perfused porous structure are highly dependent on the scaffolds' structural properties. Permeability, a factor interrelated with structural factors like porosity, pore size, pore shape etc., has been shown to affect cell proliferation, cellular activity and cellular growth [22]. By implementing the results from μCT monitoring into the CFD model its effect on porosity and eventually SS could be taken into account. However, using μCT alone, it will not be possible to assess non-mineralized ECM growth.

It is important to note that the results observed are a proof-of-concept and are only valid for SF scaffolds with the reported porosity and permeability, seeded with hMSCs of the specific batch used and cultured in the perfusion bioreactor described. For different scaffold materials, cell types, cell donors and bioreactor geometries, SS are likely to differ and have to be defined first. The experiment presented can be used in further experiments to investigate possible differences of regenerative potential of cells from different donors. CFD simulations were performed with a simplified scaffold model based on Darcy's law that is not able to display local differences in scaffold geometry. Differences observed in mineralized tissue growth between single samples (Fig 3) might be explained by local differences in scaffold geometry leading to local differences in SS and subsequent differences in mineralized tissue growth patterns. Nevertheless, the ROC analysis showed good agreement of the SS based prediction of mineralized tissue growth.

This study showed a clear dependence of hMSC fate on the perfusion velocity applied. The velocities applied were able to mimic the mechanical environment during fracture healing or in healthy bone tissue leading to increased cell number and ECM production (v_low_) or mineralized matrix growth (v_high_), respectively. Mineralized ECM formation in the static control group revealed that the production of mineralized ECM at v_low_ was suppressed. Two distinct ranges of SS could be defined by CFD showing no overlap of SS between v_low_ and v_high_. This leads to the assumption that there exist optimal SS where hMSCs enter cell proliferation or differentiation. Mineralized ECM developed from the edge of the scaffolds towards the middle. The optimal SS for mineralized ECM growth is thought to move towards the center of the scaffold due to the filling of the pores on the edge. By combining the observations from the μCT monitoring with the CFD model presented, the mechanical environment may be modeled in the future over the whole culture period. Together with the optimal SS defined, this study lays the foundation for a tight control of hMSC cell behavior towards proliferation or differentiation in perfusion cultures over the whole culture period.

## Supporting information

S1 File Zip file of all relevant data within the paper. The zip file contains results of biochemical assays (metabolic activity per cell, DNA amount, osteogenic differentiation), μCT monitoring, ROC analysis, scaffold permeability determination, SS and velocity simulations.
(ZIP)
